# Engineered biosynthesis of enduracidin lipoglycopeptide antibiotics using the ramoplanin mannosyltransferase Ram29

**DOI:** 10.1099/mic.0.000095

**Published:** 2015-07

**Authors:** Ming-Cheng Wu, Matthew Q. Styles, Brian J. C. Law, Anna-Winona Struck, Laura Nunns, Jason Micklefield

**Affiliations:** School of Chemistry and Manchester Institute of Biotechnology, The University of Manchester, 131 Princess Street, Manchester M1 7DN, UK

## Abstract

The lipopeptides ramoplanin from *Actinoplanes* sp. ATCC 33076 and enduracidin produced by *Streptomyces fungicidicus* are effective antibiotics against a number of drug-resistant Gram-positive pathogens. While these two antibiotics share a similar cyclic peptide structure, comprising 17 amino acids with an *N*-terminal fatty acid side chain, ramoplanin has a di-mannose moiety that enduracidin lacks. The mannosyl substituents of ramoplanin enhance aqueous solubility, which was important in the development of ramoplanin as a potential treatment for *Clostridium difficile* infections. In this study we have determined the function of the putative mannosyltransferase encoded by *ram29* from the ramoplanin biosynthetic gene cluster. Bioinformatics revealed that Ram29 is an integral membrane protein with a putative DxD motif that is suggested to bind to, and activate, a polyprenyl phosphomannose donor and an extracytoplasmic C-terminal domain that is predicted to bind the ramoplanin aglycone acceptor. The *ram29* gene was cloned into the tetracycline inducible plasmid pMS17 and integrated into the genome of the enduracidin producer *S. fungicidicus*. Induction of *ram29* expression in *S. fungicidicus* resulted in the production of monomannosylated enduracidin derivatives, which are not present in the WT strain. Tandem MS analysis showed that mannosylation occurs on the Hpg^11^ residue of enduracidin. In addition to confirming the function of Ram29, these findings demonstrate how the less common, membrane-associated, polyprenyl phosphosugar-dependent glycosyltransferases can be used in natural product glycodiversification. Such a strategy may be valuable in future biosynthetic engineering approaches aimed at improving the physico-chemical and biological properties of bioactive secondary metabolites including antibiotics.

## Introduction

There is an urgent need for new antibiotics to combat emerging drug-resistant pathogens. The majority of antibiotics that are used to treat infectious diseases are natural products or derivatives thereof (Wu *et al.*, 2012). Natural products often require further chemical modification, to improve their biological activities or physico-chemical properties for therapeutic applications. However, many of the most promising natural products, such as the polyketides and nonribosomal peptides, are highly complex molecules that offer limited opportunity for semisynthesis and are invariably inaccessible through total synthesis on the scale required for drug development. Consequently, alternative biosynthetic engineering approaches are required that can enable the structural diversification and optimization of promising natural product scaffolds.

Biosynthetic engineering utilizes knowledge of the structure, organization and function of biosynthetic enzymes to reprogramme biosynthetic pathways at the genetic level, to produce new and potentially improved natural products (Wilkinson & Micklefield, 2007; Wu *et al.*, 2012). Previously, we adopted biosynthetic engineering approaches to diversify the calcium-dependent lipopeptide antibiotics (CDAs), leading to a library of ‘non-natural’ CDAs (Amir-Heidari *et al.*, 2008; Lewis *et al.*, 2011; Mahlert *et al.*, 2007; Milne *et al.*, 2006; Neary *et al.*, 2007; Powell *et al.*, 2007a, b; Thirlway *et al.*, 2012; Uguru *et al.*, 2004). In this work we focus on the biosynthesis of related lipoglycopeptide antibiotics: ramoplanin from *Actinoplanes* sp. ATCC 33076 (McCafferty *et al.*, 2002), and the enduracidins produced by *Streptomyces fungicidicus* (Yin & Zabriskie, 2006).

**Fig. 1.**
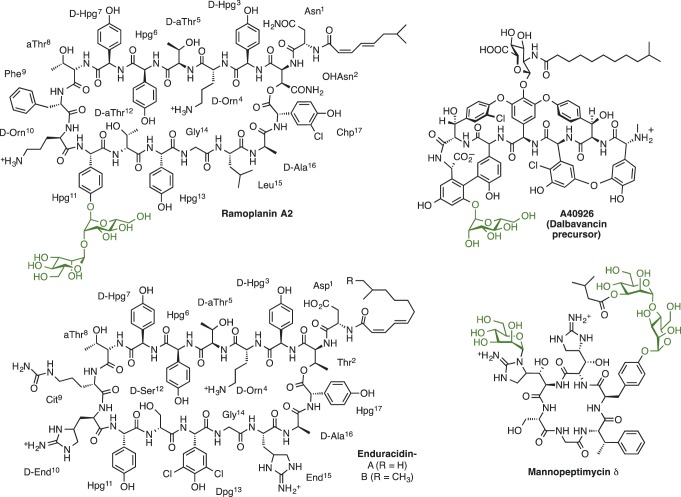
Structures of the nonribosomal peptide antibiotics enduracidin, ramoplanin, mannopeptimycin and the dalbavancin precursor (A40926). All, except enduracidin, possess mannosyl substituents (in green) derived from putative polyprenyl phosphomannose-dependent glycosyltransferases Ram29, MppH/I and Dbv20 respectively.

Ramoplanin (Fig. 1) is a highly effective antibiotic against a number of Gram-positive bacterial pathogens such as methicillin-resistant *Staphylococcus aureus* and vancomycin-resistant enterococci (McCafferty *et al.*, 2002; Walker *et al.*, 2005), and has entered phase III clinical trials for the treatment of *Clostridium difficile* infections. Enduracidin shares a similar structure with ramoplanin (Fig. 1) and is also a potent antibiotic; both antibiotics bind to lipid II, blocking the subsequent transglycosylation reactions during peptidoglycan biosynthesis of the bacterial cell wall (Cudic *et al.*, 2002; Fang *et al.*, 2006; Hamburger *et al.*, 2009). However, enduracidin has poor aqueous solubility compared with ramoplanin (Cudic *et al.*, 2002). Consequently, whilst ramoplanin has entered clinical trials, enduracidin is instead utilized as an additive in animal feed (Castiglione *et al.*, 2005; McCafferty *et al.*, 2002; Yin & Zabriskie, 2006). The main structural difference between the two antibiotics is the presence of mannosyl groups on hydroxyphenylglycine^11^ (Hpg^11^) that contribute significantly to the solubility of ramoplanin but are absent in enduracidin. The mannosyl groups of ramoplanin are not required for antibacterial activity (Cudic *et al.*, 2002) as the aglycone retains similar potency to the parent lipoglycopeptide. However the mannosyl groups of ramoplanin, in addition to enhancing solubility, contribute to the hydrolytic stability of the peptide core (Cudic *et al.*, 2002). In addition the mannosyl groups can provide a chemical handle for semisynthesis. For example, the mannosyl groups of the mannopeptimycins have been selectively derivatized to generate semisynthetic products with increased activity (He, 2005).

Within the ramoplanin (*ram*) biosynthetic gene cluster there is a gene, *ram29*, encoding an integral membrane protein, that is absent from the enduracidin (*end*) cluster. Genes encoding similar proteins are also present within the gene clusters for other important mannosylated products, including the mannopeptimycins (*mppH*/*I*) (Magarvey *et al.*, 2006), and the commercial glycopeptide antibiotics teicoplanin (*tcp15*) (Sosio *et al.*, 2004) and dalbavancin (*dbv20*) (Sosio *et al.*, 2003) (Fig. 1). The putative mannosylating enzymes have not been characterized, but are suggested to transfer mannose (Man) from a polyprenyl phosphomannose (PPM) donor within the membrane (Magarvey *et al.*, 2006). A recent report (Chen *et al.*, 2013) described the deletion of *ram29* from the ramoplanin producer, *Actinoplanes* sp. ATCC 33076. The resultant deletion strain was shown to produce the ramoplanin aglycone, suggesting that Ram29 is responsible for the incorporation of the mannose sugars in ramoplanin. In this paper, we have established conditions for the heterologous expression of *ram29* in the enduracidin-producing strain of *S. fungicidicus*, confirming the mannosyltransferase function of Ram29, and also providing access to novel mannosylated enduracidin variants.

## Methods

### Bioinformatics analysis

The topology of Ram29 was predicted using bioinformatics programs which are applied widely to membrane protein analysis, namely tmpred (Hofmann & Stoffel, 1993), hmmtop (Tusnády & Simon, 2001), tmhmm (Möller *et al.*, 2001), scampi (Bernsel *et al.*, 2008), prodiv (Viklund & Elofsson, 2004), pro (Viklund & Elofsson, 2004), octopus (Viklund & Elofsson, 2008) and topcons (Viklund & Elofsson, 2004). The DNA sequence was analysed using Vector NTI software V-10.1.1 (Invitrogen), and the blast and alignment analyses were conducted using the alignment program from the same software.

### Bacterial strains, microbiological methods and genetic manipulations

The bacterial strains *S. fungicidicus* (ATCC 31731) and *Actinoplanes* sp. (ATCC 33076) were purchased from the ATCC bioresource centre (LGC Standards) and cultured in ISP Medium 2 and ISP Medium 1 broth respectively (Shirling & Gottlieb, 1966). Prior to preparation of spore stocks, both strains were cultured on mannitol soya (MS) flour media for 2 weeks (Hobbs *et al.*, 1989). Mycelia from 1 ml of culture were used for genomic DNA extraction using a Qiagen Genomic DNA extraction kit (Qiagen) following the manufacturer's protocol for bacterial genomic DNA extraction. The genomic DNA was redissolved in 100 μl of 10 mM Tris/HCl buffer pH 8.5 and used as a template for PCR. The gene-specific primers ram29-F1, ram29-R1, ram29-F2 and ram29-R2 (Table S1, available in the online Supplementary Material) were used to amplify the *ram29* sequence. The resultant PCR product was digested with *Bam*HI alone or doubly digested with *Eco*RV and *Nsi*I and cloned into the similarly digested pIJ86 and pMS17 to yield pIJ86-*ram29* and pMS17-*ram29* (Fig. S1). pIJ86 is a non-integrative vector with a constitutive ermE* promoter which has been widely used for protein expression in *Streptomyces* spp. (Bibb *et al.*, 1985). pMS17 is a conjugative and integrative plasmid containing a tetracycline (Tc)-inducible promoter (kindly provided by Professor M. Smith, University of York) (Rodríguez-García *et al.*, 2005; Wehmeier *et al.*, 2009). *Escherichia coli* TOP10 (Invitrogen) was used for cloning procedures. The integrity of pIJ86-*ram29* and pMS17-*ram29* was confirmed by DNA sequencing (GATC Biotech) prior to being transformed into the dam, dcm and hsdM methylation-deficient *E. coli* strain ET12567 carrying the non-transmissible pUZ8002 (MacNeil *et al.*, 1992). Intergeneric conjugation was carried out between *E. coli* ET12567/pUZ8002 harbouring either pIJ86-*ram29* or pMS17-*ram29* and *S. fungicidicus* (Kieser *et al.*, 2000). Following 3–6 days of incubation at 30 °C exconjugants were transferred to selective MS agar plates supplemented with apramycin (100 μg ml^− 1^) and nalidixic acid (25 μg ml^− 1^). Following 2 weeks of incubation at 30 °C, spores derived from the exconjugants were transferred to bouillon broth (1 % meat extract, 1 % peptone, 0.5 % NaCl, 0.2 % K_2_HPO_4_, pH 7.0; modified from ATCC Medium 841) and incubated at 30 °C for 2–4 days. The mycelia were pelleted by centrifugation and the genomic DNA extracted for use in sequence confirmation.

### Isolation of enduracidin from *S. fungicidicus* fermentation media

The spores of WT or exconjugant *S. fungicidicus* strains (10^5^–10^7^) were used to inoculate 75 ml of seed medium (Bouillon broth) and cultured for 3 days at 30 °C. Ten millilitres of seed culture was then transferred to 300 ml of enduracidin fermentation medium (Nogami *et al.*, 1984) supplemented with the appropriate antibiotics and cultured at 30 °C with shaking for 14–21 days. For exconjugants harbouring pMS17-*ram29,* tetracycline was added to a final concentration of 0.1 μg ml^− 1^ every 3 days. Following fermentation, cultures were transferred to 50 ml Falcon tubes and the mycelia pelleted by centrifugation (15 min, 10 000 ***g***). Pellets were resuspended in 70 % acidic methanol (pH 2.0) and stirred for 3 h. Supernatants of the methanol extracts were obtained by centrifugation (10 min, 10 000 ***g***) and proteins and lipids extracted with an equal volume of ethyl acetate. Following separation, the pH of the aqueous partition was adjusted to 8.2 and the enduracidin extracted using 300 ml (3 × 100 ml) of butanol. The butanol phase, containing enduracidin, was washed with water (3 × 100 ml), and then the enduracidin was partitioned into 300 ml (3 × 100 ml) of 5 mM HCl aqueous solution (pH 2.0). The pH of the aqueous layer was adjusted to 8.2 and the enduracidin extracted with 300 ml (3 × 100 ml) of butanol. The butanol layer containing enduracidin was separated and concentrated by evaporation under reduced pressure, leaving a crude white powder, which was redissolved in 20 % acetonitrile. Enduracidin was further purified by reverse-phase high-performance liquid chromatography (RP-HPLC) using a Gemini C18 column (10 × 250 mm, 5 μm; Phenomenex) using a gradient composed of water with 0.1 % formic acid (A) and acetonitrile with 0.1 % formic acid (B) as follows: 10 min linear gradient from A/B (95 : 5 %) to A/B (80 : 20 %), 30 min linear gradient to A/B (60 : 40 %), 5 min linear gradient to A/B (5 : 95 %), held at A/B (5 : 95 %) for 4 min, 3 min linear gradient to A/B (95 : 5 %) before re-equilibration at A/B (95 : 5 %) over 8 min. A flow rate of 3 ml min^− 1^ was used and elution of enduracidin was monitored at A_230_. Fractions were collected and further analysed by liquid chromatography (LC)-MS.

### Analysis of enduracidin extracts using LC-MS

LC-MS analysis was carried out on a Micromass LCT TOF mass spectrometer (Waters), equipped with an electrospray ionization source run in positive mode (scanning from *m*/*z* 700 to 2500) combined with a Waters 2790 separation module. Samples were dissolved in acetonitrile/water/formic acid (50/50/0.1). HPLC was carried out using a Gemini C18 column (4.6 × 150 mm, 3 μm; Phenomenex) at a flow rate of 1 ml min^− 1^ using a gradient composed of water with 0.1 % formic acid (A) and acetonitrile with 0.1 % formic acid (B) as follows: 10 min linear gradient from A/B (80 : 20 %) to A/B (30 : 70 %), 1 min gradient to A/B (5 : 95 %) and held at A/B (5 : 95 %) for further 4 min, before re-equilibration at A/B (80 : 20 %) for 4 min.

### Tandem MS

Tandem MS analysis was carried out on a Q-TOF Ultima Global instrument (Waters) by direct infusion of a purified sample dissolved in acetonitrile/water/formic acid (50/50/0.1). Borosilicate nanoemitters (Proxeon) were loaded with sample using gel loading pipette tips. The nanoemitters were mounted onto the source and then activated at 0.4 kV capillary voltage by applying pressure against the sample cone, snapping the glass fibre. Once activated, the capillary voltage was ramped steadily until a consistent ion beam was observed, typically between 1.6 and 1.8 kV. Each nanoemitter holds approximately 20 μl and provides a spray for approximately 30 min duration, giving a flow rate of approximately 0.66 μl min^− 1^. The instrument was calibrated using the tandem mass spectrum of [Glu]-Fibrinopeptide B.

Once an ion beam was achieved, the MS source was tuned to the product by ramping the capillary voltage and the cone voltage until the signal intensity peaked. An MS scan was carried out to determine a suitable *m*/*z* value to fragment. An MS-MS scan was then carried out by selecting the triply charged [M+3H]^3+^ ion (785 for the WT enduracidin and 839 for the mannosylated enduracidin) as the parent ion for analysis, and raising the collision energy within the collision cell until the parent ion was a minority peak on the resulting spectrum. The data were collected over 20 min, or until a sufficient spectrum was achieved for sequencing analysis. The subsequent spectrum was background subtracted (polynomial order = 4, 40 % below curve, tolerance = 0.010), smoothed (Savitzky Golay smoothing, smooth channels = ± 5, number of smooths = 2), and centred (minimum peak width at half height = 4, 80 % centroid top) by the MassLynx software (Version 4.0, Waters Ltd) prior to analysis.

### Enduracidin sequencing

A database of theoretical *m*/*z* values was set up based on all the possible fragmentation patterns of enduracidin, assuming two cleavages yielding a linear peptide ion. Additionally, a further database was set up assuming that the first cleavage occurs at the ester bond linkage; thus additional fragmentation events yielded *a*, *b*, *y* and *z* ions at either one or both ends (as both the C- and N-termini have undergone fragmentation). Tandem MS data were then compared with these theoretical values to generate ion series that can be used to characterize the peptide, and signature losses were identified by observation.

## Results and Discussion

### Bioinformatics and proposed mannosylation mechanism

**Fig. 2.**
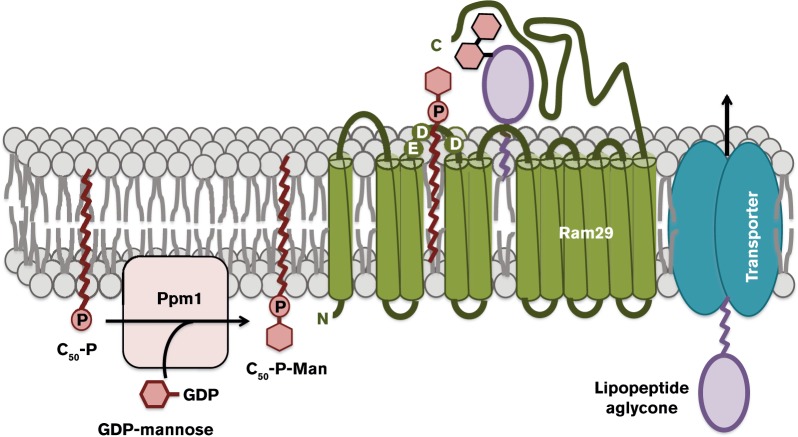
Proposed two-component system for mannosylation of the glycopeptide antibiotics. PPM synthase (Ppm1) is located in the membrane and catalyses the synthesis of PPMs on the cytoplasmic membrane surface, before being translocated to the extracytoplasmic membrane surface. Subsequently, the membrane protein mannosyltransferase Ram29 transfers the mannose moiety from PPM to the antibiotic aglycone, which has been transported from the cytoplasm.

Sequence similarity searches predict that Ram29 is an integral membrane protein, from the GT-C superfamily of glycosyltransferases, which is likely to be similar to other predicted mannosyltransferases including MppH/I (Magarvey *et al.*, 2006), Tcp15 (Sosio *et al.*, 2004) and Dbv20 (Sosio *et al.*, 2003). Ram29 was further analysed using eight bioinformatics programs designed to predict the topology of membrane proteins. Depending on the algorithm used, Ram29 is predicted to possess between 10 and 14 transmembrane segments (TMS) spanning approximately 450 amino acids within the N-terminal region of the protein. Although the bioinformatics algorithms did not converge on the precise number of TMS, all of these programmes predict that the C-terminal domain, comprising approximately 150 amino acids, is extracytoplasmic. We suggest that this putative extracytoplasmic C-terminal domain, which shows little or no similarity with any other known protein sequences, is responsible for binding the ramoplanin aglycone (Figs. 2 and S2). This is consistent with earlier findings (Borghi *et al.*, 1991) showing that the demannosylated teicoplanin aglycone can be remannosylated by incubating with the teicoplanin producing strain, possessing the putative mannosyltransferase Tcp15, demonstrating that mannosylation occurs on the outside of the cell membrane.

Other members of GT-C superfamily possess similar TMS, with acidic (DxD or DDx) motifs in either the first or second extracytoplasmic loop (Berg *et al.*, 2005; Liu & Mushegian, 2003). The DxD motif is suggested to bind to, and activate, polyprenyl phosphosugar donors (Berg *et al.*, 2007), catalysing transfer of a range of different sugars, including glucose (Glu) (Song *et al.*, 2009), galactosamine (GalN) (Skovierová et al., 2010), arabinofuranose (Araf) (Berg *et al.*, 2005) and rhamnose (Rha) (Birch *et al.*, 2009), during cell wall oligosaccharide biosynthesis. Sequence alignments of Ram29 led to the identification of a putative DxD motif in the second predicted extracytoplasmic loop, containing acidic residues (E162, D163 & D175) which are conserved in other putative mannosyltransferases (Tcp15 and Dbv20) (Fig. S3). Taken together, we suggest that Ram29 would use its unique C-terminal domain to recognize and bind the ramoplanin aglycone outside of the cell membrane, and the extracytoplasmic loop containing the DxD motif would recognize and transfer the mannosyl group from PPMs, which are known to be biosynthesized from GDP-mannose and polyprenyl phosphates, of varying chain length, by the PPM synthases (Ppm1) (Wehmeier *et al.*, 2009) to form mannosylated antibiotics (Fig. 2).

### Optimizing expression of *ram29* in the enduracidin producer *S. fungicidicus*

The *in vitro* characterization of the putative mannosyltransferase Ram29, like other members of the GT-C superfamily enzymes, is complicated by difficulties associated with the overproduction, isolation and purification of membrane proteins as well as the lack of available PPM donor substrates with the required polyprenyl chain length. In light of this, we chose to establish the heterologous expression system for *ram29* in the enduracidin producer *S. fungicidicus*. Given the structural similarity of enduracidin to the ramoplanin aglycone, we predicted that the endogenous enduracidin and PPM might function as substrates for Ram29 leading to mannosylated enduracidins. In addition to confirming the function of Ram29, this approach could potentially provide new mannosylated enduracidin antibiotics with improved properties, particularly those with enhanced aqueous solubility.

Accordingly, an expression cassette with the putative mannosyltransferase encoding gene *ram29,* including its native upstream Shine–Dalgarno (SD) sequence, was cloned into the high copy number plasmid pIJ86 under the control of the strong constitutive promoter ermE* (Bibb *et al.*, 1985). The LC-MS analysis of cellular extracts and culture supernatants derived from *S. fungicidicus* pIJ86-*ram29* exconjugants failed to lead to the detection of any mannosylated enduracidins. Given that Ram29 is predicted to be an integral membrane protein, the higher levels of expression afforded by pIJ86 could result in production of insoluble, non-functional Ram29. Consequently *ram29* was placed under the control of a tetracycline inducible promoter by cloning the expression cassette into the integrative plasmid pMS17 (Rodríguez-García *et al.*, 2005; Wehmeier *et al.*, 2009). However, even with inducible control over the expression of the *ram29* gene integrated onto the chromosome of *S. fungicidicus*, we still failed to detect any mannosylated enduracidin, which we rationalized may be due to a suboptimal sd sequence and GTG start codon of *ram29*. We therefore replaced the 5′-leader sequence of *ram29*, including the native sd sequence and start codon, with the corresponding sequence of an eGFP expression construct from the plasmid pIJ8668 (kindly provided by Professor M. Bibb, John Innes Centre), which gives robust expression of eGFP (Fig. S4). The resulting *S. fungicidicus* pMS17-*ram29* exconjugants were shown to produce mannosylated enduracidins (*vide infra*), demonstrating the importance of the sd sequence and the distance between the sd sequence and the alternative ATG start codon for establishing functional expression of *ram29*.

### Isolation of mannosylated enduracidins from *S. fungicidicus* pMS17-*ram29*

**Fig. 3.**
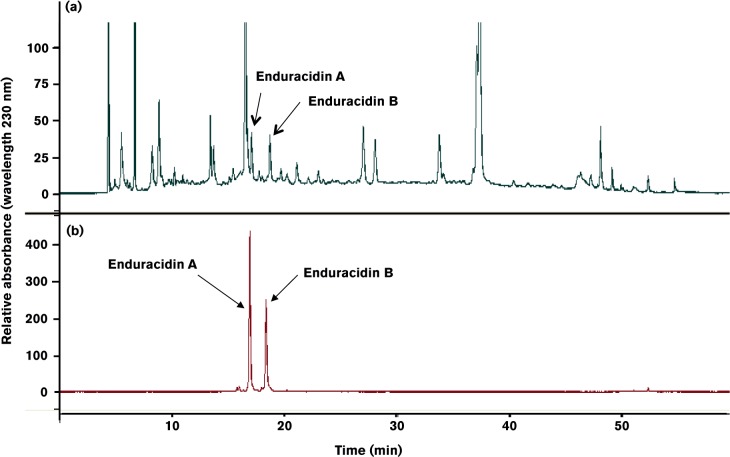
RP-HPLC analysis of the *S. fungicidicus* crude extract. (a) Crude extract from the fermentation broth of the *S. fungicidicus* pMS17-*ram29* transformant. (b) Enduracidin A and B standards.

**Fig. 4.**
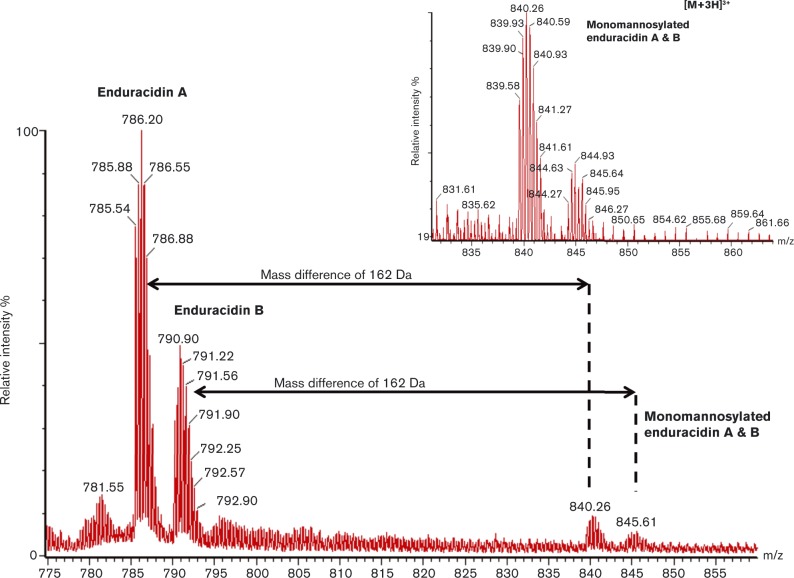
ESI (electrospray ionisation)-LC-MS analysis of the *S. fungicidicus* pMS17-*ram29* fermentation products. The spectra show the triply charged [M+3H]^3+^ parent ions for WT enduracidins A (*m*/*z*: observed 785.5; expected 785.3) and B (*m*/*z*: observed 790.2; expected 790.0) and monomannosylated enduracidins A (*m*/*z*: observed 839.6, expected 839.3) and B (*m*/*z*: observed 844.3, expected 844.0). Doubly charged [M+2H]^2+^ molecular ions were also observed by ESI-LC-MS (Fig. S5), and the singly charged [M+H]^+^ ions were observed by MALDI-TOF (Fig. S6), consistent with the proposed structures.

The optimized *S. fungicidicus* pMS17-*ram29* was grown in liquid media for 2 weeks, the enduracidin isolated using a butanol extraction procedure and further purified by RP-HPLC (Fig. 3). Fractions containing enduracidin were identified by MALDI-TOF (Fig. S5) and further analysed by LC-MS (Fig. 4). It was revealed that the strain *S. fungicidicus* pMS17-*ram29* produced two additional new minor products differing in mass by +162 Da to enduracidins A and B, which were also evident. The +162 Da mass difference is consistent with the addition of a single mannose group to enduracidins A and B. The two new putative mannosylated enduracidin A and B derivatives were absent from extracts derived from WT *S. fungicidicus* fermentations.

**Fig. 5.**
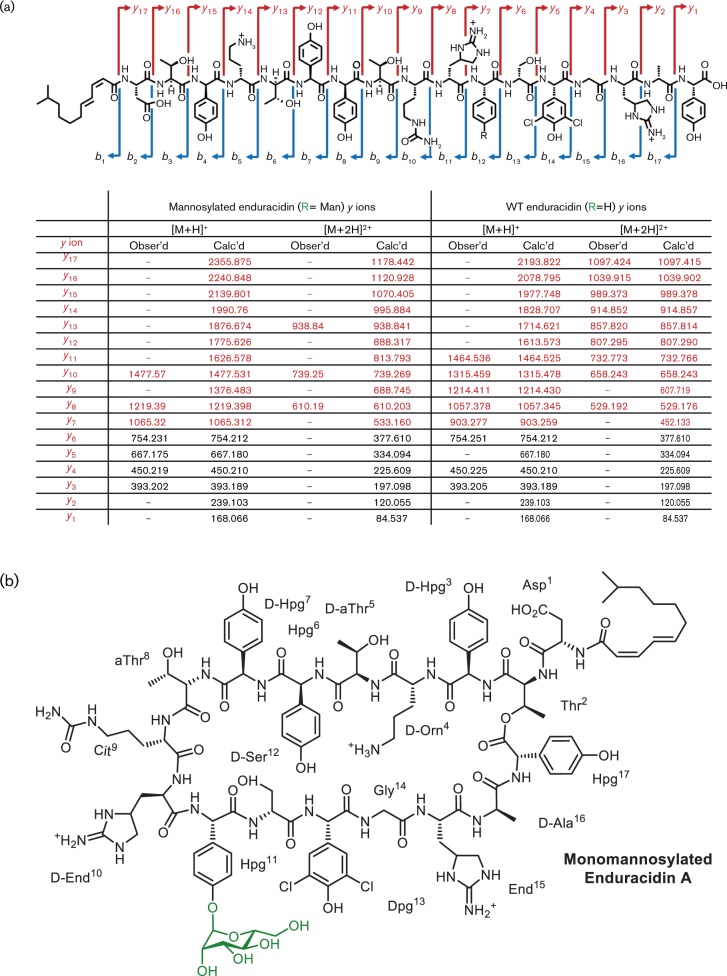
Tandem MS of WT and monomannosylated enduracidin A. (a) Localization of the mannosyl group on monomannosylated enduracidin A by MS-MS characterization. The *y* ions highlighted in red denote a mass difference of +162 Da from WT to mannosylated. The key *y*_7_ ion is the last fragment to carry the +162 mannosyl group mass. (b) Structure of the monomannosylated enduracidin A analogue, based on MS-MS data. Additional MS-MS data (Tables S2–S5) and spectra (Figs S7 and S8) supporting the structure of monomannosylated enduracidin A can be found within the supplementary information.

The position of the putative mannose group was determined by tandem MS. The *de novo* sequencing of cyclic nonribosomal peptides can be non-trivial, as each linear fragment is the product of at least two fragmentation events, and the subset of possible fragments is dependent on the position of the first event. By running a series of experiments on both enduracidin A and the new glycosylated analogue in parallel, it was possible to deduce a *y*-ion series for both molecules under identical conditions. In the spectra, a prominent *y*-ion series can be observed for the WT enduracidin A peptide fragments: End^15^–Hpg^17^ (*y*_3_, *m*/*z* 393.2), Gly^14^–Hpg^17^ (*y*_4_, *m*/*z* 450.2), Ser^12^–Hpg^17^ (*y*_5_, *m*/*z* 754.3), Hpg^11^–Hpg^17^ (*y*_7_, *m*/*z* 903.3), End^10^–Hpg^17^ (*y*_8_, *m*/*z* 1057.4), Cit^9^–Hpg^17^ (*y*_9_, *m*/*z* 1214.4), Thr^8^–Hpg^17^ (*y*_10_, *m*/*z* 1315.5) and Hpg^7^–Hpg^17^ (*y*_11_, *m*/*z* 1464.5) (Fig. 5). In contrast, analysis of the monomannosylated enduracidin peptide revealed new fragment ions with *m*/*z* corresponding to: man–Hpg^11^–Hpg^17^ (*y*_7_, *m*/*z* 1065.3), End^10^–Hpg^17^ (*y*_8_, *m*/*z* 1219.4), Thr^8^–Hpg^17^ (*y*_10_, *m*/*z* 1477.5) (Fig. 5). Comparison of the key observed ions *y*_7_, *y*_8_, and *y*_10_ shows a mass difference of +162 Da between the WT and the mannosylated sample, corresponding to the addition of a single mannose group in the latter. Additional ions corresponding to non-hydrated fragment ions, and doubly charged fragments ions (both hydrated and non-hydrated) are described in the supplementary information (Tables S2–S5, Figs S7 and S8). The MS-MS spectra provide strong evidence as to the regioselectivity of Ram29, with the mannosylation occurring on the Hpg^11^ residue of enduracidin: the +162 Da mass difference was carried throughout the mannosylated peptide sample from *y*_10_ to *y*_7_; however once Hpg^11^ was lost, thus generating the *y*_6_ ion, this and all subsequent fragment ions were identical with those of the WT enduracidin (Fig. 5). Thus, Ram29 appears to mannosylate enduracidin at the same position as on ramoplanin. Moreover, Ram29 is evidently promiscuous and can mannosylate alternative lipopeptides to ramoplanin, such as the enduracidins A and B.

The deletion of *ram29* from the ramoplanin producer, *Actinoplanes* sp. ATCC 33076 (Chen *et al.*, 2013), was shown to produce the ramoplanin aglycone which was consistent with the findings presented here. Given that Ram29 is the only apparent glycosyltransferase in the ramoplanin biosynthetic gene cluster, this suggests that Ram29 may act iteratively to introduce both the mannosyl groups of ramoplanin (Chen *et al.*, 2013). Interestingly, we show that expression of Ram29 in *S. fungicidicus* results in only monomannosylated enduracidins. Thus whilst Ram29 clearly recognizes the phenol group of Hpg^11^ in both enduracidin and the ramoplanin aglycone as its acceptor substrate, it is possible that a second mannosyltransferase, which is not encoded from within the ramoplanin biosynthetic gene cluster, may be required to introduce a second distinct α1,2-dimannosyl glycosidic linkage. Alternatively, some *Streptomyces* species have been reported to have membrane-associated α-mannosidases which remove mannosyl groups from antibiotics. For example, the mycelia of *Streptomyces* GE 91081 can remove one mannose unit from ramoplanin (Di Palo *et al.*, 2007), whilst the mycelia of *Nocardia orientalis* NRRL 2450 and *Streptomyces candidus* NRRL 3218 have been found to remove a mannose unit from teicoplanin (Borghi *et al.*, 1991). *S. fungicidicus* contains a putative mannosidase encoding gene (*orf13*) within the enduracidin gene cluster (Yin & Zabriskie, 2006), which may function to remove a mannose group from any dimannosylated enduracidin that may be produced. Mannosidase activity could also potentially account for the low yields of mannosylated enduracidins produced by the *S. fungicidicus* pMS17-*ram29* strain.

## Conclusion

In summary, we have developed protocols for the functional expression of the mannosyltransferase Ram29 in the enduracidin producer *S. fungicidicus* and isolated novel monomannosylated enduracidin variants. Using tandem MS, we have shown that mannosylation occurs at Hpg^11^ of enduracidin, which supports the function of Ram29 in the biosynthesis of ramoplanin. Previously there have been considerable advances in the development of natural product glycodiversification strategies, which have largely focused on glycosylation pathways that involve the more common Leloir-type (sugar nucleotide-dependent) glycosyltransferases. The findings presented here provide the first example of how the less common, membrane associated PPM-dependent glycosyltransferases can also be used in glycodiversification of natural product scaffolds; this could be valuable in future efforts to develop bioactive natural products, including improved enduracidin variants with enhanced aqueous solubility.
